# Light-guided dynamic phantom to mimic microvasculature for biomedical applications: an exploration for pulse oximeter

**DOI:** 10.1117/1.JBO.29.S3.S33312

**Published:** 2024-12-18

**Authors:** Hui Ma, Dario Angelone, Claudia Nunzia Guadagno, Stefan Andersson-Engels, Sanathana Konugolu Venkata Sekar

**Affiliations:** a Tyndall National Institute, Biophotonics@Tyndall, Cork, Ireland; b University College Cork, School of Engineering Science, Cork, Ireland; c University College Cork, School of Physics, Cork, Ireland; d BioPixS Ltd., Cork, Ireland

**Keywords:** dynamic phantom, pulse oximeter, photoplethysmography, standardization, near-infrared spectroscopy, diffuse optics

## Abstract

**Significance:**

Dynamic phantoms capable of changing optical properties by control are essential for standardizing and calibrating spectroscopy systems such as the pulse oximeter. However, current liquid dynamic phantoms containing human blood have a short shelf life and require complex experimental setups. Some solid dynamic phantoms are influenced by the angular-dependent performance of the liquid crystal display (LCD), some have a low spatial resolution, and some have slow control of optical properties.

**Aim:**

We aimed to develop a solid dynamic phantom, which can overcome these obstacles by changing the optical properties rapidly and generating dynamic biological signals.

**Approach:**

The absorption properties of the phantom can be controlled in real time by modulating an LCD. A light guide was employed to avoid the angular-dependent performance of the LCD by isolating the scattering top-layer tissue-mimicking silicone phantom from the LCD.

**Results:**

The dynamic phantom was characterized at 940, 660, 530, and 455 nm to create a lookup table. Photoplethysmography signals of different heart rates from 80 to 120 beats per minute were synthesized, and oxygen saturation levels at 86%, 90%, 95%, and 100% were generated at multiple wavelengths.

**Conclusions:**

The design, characterization, and potential applications of the dynamic phantom have been presented. This dynamic phantom can simulate various biological signals by applying corresponding modulation signals and has the potential to calibrate and validate pulse oximeter, imaging, and spectroscopy systems.

## 1 Introduction

Biomedical sensing and imaging play a crucial role in modern clinical analysis, providing visualizations of body tissues, facilitating non-invasive or minimally invasive diagnostic information, and offering real-time surgical guidance.^1^^,^^2^ It helps to reduce the time and cost of diagnosis and treatment and improve patient satisfaction. However, the lack of standards for evaluating devices and comparing systems across the field hampers both technological advancement and the clinical translation of optical sensing and imaging systems.^3^^,^^4^ Hemoglobin concentration in human blood is an important parameter to monitor the physiological condition. It gives information on the capability of oxygen transportation in blood, provides preoperative and postoperative care, helps to diagnose diseases such as anemia, evaluates treatment response, and assesses the performance of athletes.^5^^–^^10^ With the emergence of portable and wearable health monitoring devices, there is an increasing demand for optical standards capable of dynamically changing their optical properties to replicate changes in hemoglobin oxygen saturation during the respiratory and heart-beat cycle. Biophotonics phantoms are artificial structures designed to simulate the optical properties of biological media. They are essential tools for simulating light distribution with the geometry of physical tissues, validating and characterizing the performance of optical devices, and recording reference measurements.^11^ Many different phantoms with high stability, controllability, and repeatability have been developed for device calibration, optimization, and quality control.^12^^–^^16^

Researchers have developed liquid phantoms using whole blood or extracted erythrocytes to mimic changes in oxygen saturation. However, the use of blood components could raise ethical and safety concerns and limit the stability and shelf life of the phantoms. In addition, biological variability in blood components reduces the reproducibility of the phantom. To maintain the appropriate conditions, temperature and pH must be monitored throughout the entire measurement process. The use of temperature and pH monitors, peristaltic pumps, and membrane oxygenators increases the cost and complexity of the experimental setup. Oxygen saturation levels are modulated by adding oxygenating compounds such as oxygen and deoxygenating compounds such as yeast or nitrogen bubbling or a mixture of d-glucose, glucose oxidase, and catalase.^17^^–^^23^ A peristaltic pump is used for blood circulation, and a membrane oxygenator is applied to control blood oxygenation. Yeast takes 20 to 30 min to gradually deoxygenate the phantom,^21^ and nitrogen bubbling takes 30 min to effectively deoxygenate the phantom,^22^ which limits the potential for real-time control.

Several solid dynamic phantoms have been developed to control changes in the absorption coefficient with high stability and reproducibility. Koh et al.^24^ designed a dynamic phantom with a liquid-crystal display (LCD) sandwiched between the solid tissue-mimicking phantom. The absorption at each pixel is electronically controlled to be on or off state. Funane et al.^25^ developed a phantom with two stage-driven absorber layers to realize gradual absorption changes by tuning the position of the absorber. Hebden et al.^26^ proposed a phantom with embedded targets comprising a black thermochromic pigment. The pigment changes from black to white when heated to 47°C. However, the performance of the LCD-based dynamic phantom was largely influenced by the scattering properties of the top layer phantoms because the attenuation of the LCD is angular dependent. The phantom with moving stages only creates macroscopic changes in tissue, and it lacks the potential to simulate complex structures such as vascular systems. For the thermochromic dynamic phantom, the smallest spacing between targets is 11.2 mm to avoid crosstalk from the neighboring target, thereby limiting the spatial resolution. In addition, it takes ∼70  s to change the color from room temperature, which is not appropriate for fast control.

In this work, we implemented a novel light guide-based dynamic phantom to overcome the challenges mentioned above.^27^ This phantom utilized a polymer optical fiber to isolate the LCD from the top-layer tissue-mimicking phantom and avoid angular-dependent performance. The absorption was tuned gradually by applying different voltages to the LCD. The phantom was characterized at four wavelengths: 455, 530, 660, and 940 nm, covering both visible and near-infrared ranges. As case studies, photoplethysmography (PPG) signals of different heart rates and oxygen saturation levels were generated by the dynamic phantom and tested by a photodiode. This dynamic phantom can be a helpful tool for calibrating and validating pulse oximeters, imaging systems, and spectroscopy devices.

## 2 Method and Material

### 2.1 Phantom Design

**Fig. 1:**
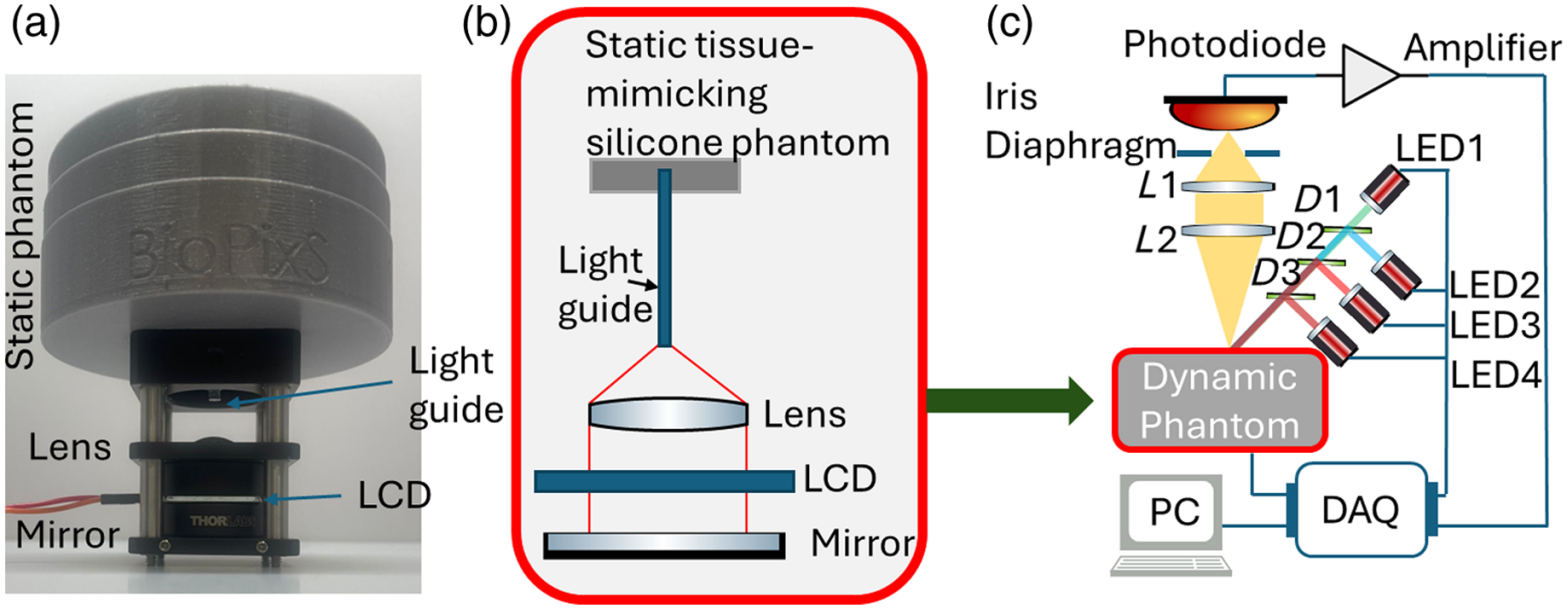
(a) Photo of the dynamic phantom. (b) Scheme of the dynamic phantom. (c) Scheme of the validation system.

Figure 1(a) shows the photo of the dynamic phantom. Figure 1(b) illustrates the schematic layout of the dynamic phantom. The light passing through a static tissue-mimicking silicone phantom was collected by a 4-cm long 3-mm diameter polymer optical fiber (OMPF3000, The Optoelectronic Manufacturing Corporation Ltd., Redruth, United Kingdom) and collimated by a condenser lens (ACL2520U, Thorlabs, Newton, New Jersey, United States) to avoid the angular dependent performance of the LCD. The parallel beam was modulated by the LCD (FOS-NIR (1100), LC-Tec Displays AB, Borlänge, Sweden) and then reflected through the phantom by a silver mirror (PF10-03-P01, Thorlabs). The LCD was controlled by a National Instruments data acquisition (NI DAQ) (USB-6212, National Instrument, Austin, Texas, United States). The thickness and optical properties of the static superficial silicone phantom can vary depending on the application. In our demonstration, a 9-cm diameter, 0.5-mm-thick round shape silicone phantom was used based on the recipe described in Ref. 15. Briefly, the silicone phantom consisted of SiliGlass (MB Fibreglass, Newtownabbey, United Kingdom) as bulk material, polycraft black silicone pigment (MB Fibreglass, UK) as absorber, and silica microspheres (440345, Sigma-Aldrich, St. Louis, Missouri, United States) as a scatterer. The optical properties of this silicone phantom were characterized by a time-domain diffuse optical spectrometer.^28^^,^^29^ The target absorption coefficient ($\mu_{a}$) was $0.1\;{\mathrm{cm}}^{-1}$, and the reduced scattering coefficient ($\mu_{s}^{\prime }$) was $10\;{\mathrm{cm}}^{-1}$.

### 2.2 Phantom Characterization System

The full dynamic phantom was further characterized by an in-house system described in Fig. 1(c). The illumination part of the system consisted of four light-emitting diodes (LEDs) at 455, 530, 660, and 940 nm (LED1: M530L4, LED2: M455L4, LED3: M660L4, and LED4: M940L4, Thorlabs). Dichroic mirrors [D1: 490-nm longpass dichroic mirror (DMLP490R, Thorlabs), D2: 605-nm shortpass dichroic mirror (DMSP605R, Thorlabs), D3: 805-nm shortpass dichroic mirror (DMSP805R, Thorlabs)] were employed to combine these LEDs into one single beam. The illumination wavelengths were selected by switching on or off any LED or any combination of LEDs. The reflected light from the phantom above the light guide was collected by a two-lens system (L1: AC254-050-AB-ML, L2: LB1676-ML, Thorlabs) and measured by a photodiode (S3590-08, Hamamatsu Photonics, Shizuoka, Japan) with an amplifier (DLPCA-200, FEMTO Messtechnik GmbH, Berlin, Germany) and fed into the data acquisition (DAQ) (USB-6212, National Instrument).

### 2.3 Dynamic Signal Synthesis

**Fig. 2:**
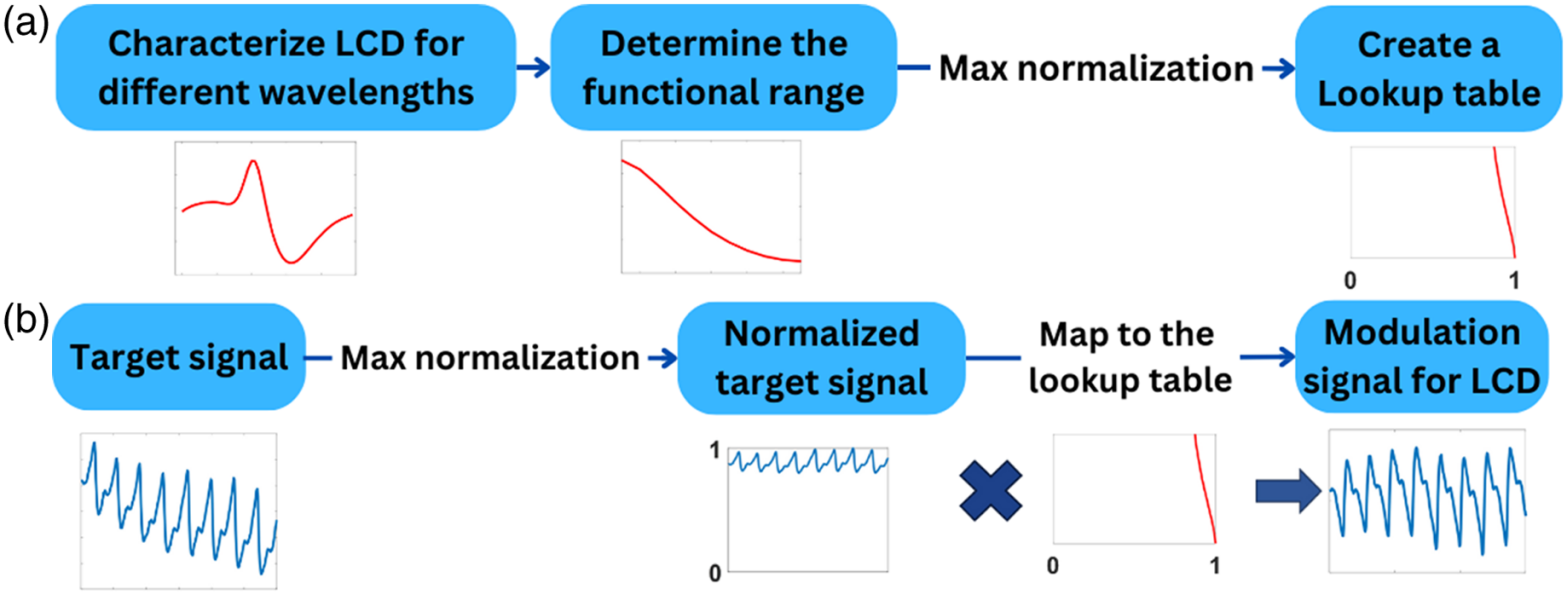
(a) Flowchart of dynamic phantom characterization. (b) Flowchart of modulation signal synthesis.

The process flow of phantom calibration is schematically described in Fig. 2(a). The intensity of the diffusely reflected light from the dynamic phantom was characterized by applying a voltage over the LCD from 0.5 to 3 V with a 0.05 V step size. The range in which this intensity was negatively correlated to the applied voltage was determined as the functional range. The response of this functional range was normalized to the maximum value to create a lookup table. The flowchart of the synthesis of the target signal is illustrated in Fig. 2(b). The target signal was also normalized to its maximum value to allow it to be mapped to the lookup table to get the modulated signal for the dynamic phantom.

### 2.4 Photoplethysmography Simulation

Two application case studies were implemented to demonstrate the dynamic phantom’s ability to reproduce different PPG signals at multiple wavelengths. One case study focused on generating PPG signals at various heart rates, whereas the other showcased the generation of PPG signals at different oxygen saturation levels.

For the heart rate simulation case study, the target synthetic PPG signals with heart rates ranging from 80 to 120 beats per minute (bpm) in steps of 10 bpm were generated using the Python toolbox NeuroKit2.^30^ Each PPG signal contains 20,000 data points (20 s) sampled at 1000 Hz. The phantom modulation voltage signals were generated by mapping synthetic PPG signals to the lookup table, as described in Sec. 2.3. The dynamic phantom was modulated and measured by the phantom characterization system as described in Sec. 2.2 at 50-Hz sampling rate. The measured signals were then processed by NeuroKit2 to recover the heart rate.^30^

For the oxygen saturation case study, the wavelengths 660 and 940 nm were selected for the characterization to enable an evaluation of different oxygen saturation levels as these wavelengths are commonly used in commercial medical pulse oximeters. A single measured PPG signal recording of 6 s from a healthy volunteer^31^ was used. It was normalized to the maximum value. Targeted signal traces for two selected wavelengths and five tested blood oxygenation levels were then generated from this normalized single trace by adjusting the modulation depth according to the blood attenuation.

These traces were then mapped to the lookup table to generate the modulated voltage signal for the dynamic phantom input. The phantom was measured by using the phantom characterization system as described in Sec. 2.2 at a 50-Hz sampling rate. The oxygen saturation can be obtained from measured PPG signals at these wavelengths through a double ratio of the pulsatile and non-pulsatile components of the measured red and near-infrared light^32^^,^^33^

$$R=\frac{\ln\;\frac{I_{\max}(\lambda_{660})}{I_{\min}(\lambda_{660})}}{\ln\;\frac{I_{\max}(\lambda_{940})}{I_{\min}(\lambda_{940})}},$$ (1)

where $I_{\max}(\lambda_{660})$ is the maximum amplitude of the pulse at 660 nm, $I_{\min}(\lambda_{660})$ is the minimum amplitude of the pulse at 660 nm, $I_{\max}(\lambda_{940})$ is the maximum amplitude of the pulse at 940 nm, and $I_{\min}(\lambda_{940})$ is the minimum amplitude of the pulse at 940 nm.

In the region of 86% to 100%, oxygen saturation (${\mathrm{SpO}}_{2}$) shows a linear correlation with the R rate^34^

$${\mathrm{SpO}}_{2}=-24.87R+113.8.$$ (2)

The R rate can thus simply be calculated by

$$R=(113.8-{\mathrm{SpO}}_{2})/24.87.$$ (3)

The linear range was simulated in this case study. The R rate was calculated at every pulse, and then, the values were averaged over a 6-s sample. Target signals of different wavelengths and oxygen saturation levels were measured one by one. The relative change in the absorption coefficient of the arterial component of blood at 660 nm is 130% when changing from 100% to 86% oxygen saturation level, whereas at 940 nm, the relative absorption coefficient variation is only 6%.^35^ As the extinction coefficient of blood does not change significantly from 86% to 100% ${\mathrm{SpO}}_{2}$ at 940 nm, we kept the amplitude constant at 940 nm and modulated the amplitude at 660 nm to simulate ${\mathrm{SpO}}_{2}$ level at 86%, 90%, 95%, and 100%.

## 3 Results

### 3.1 Dynamic Phantom Characterization

**Fig. 3:**
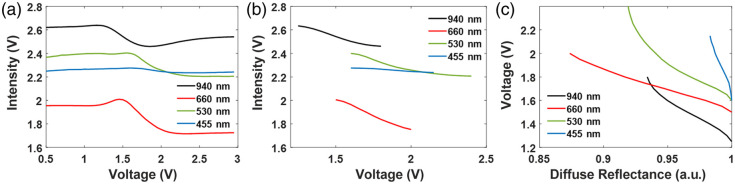
(a) Characterization results of the dynamic phantom. (b) Characterization results of the dynamic phantom in the functional range. (c) Lookup table for the dynamic phantom.

The results of the full dynamic phantom characterization are presented in Fig. 3(a). Here, the diffusely reflected light intensity from the phantom was measured at four wavelengths as a function of the voltage applied over the LCD. Within a certain voltage range (1.2 to 2.4 V), this intensity was negatively correlated to the applied voltage. This voltage range was identified as the functional range, as illustrated in Fig. 3(b). The lookup tables formed after normalizing the curve within the functional range for each wavelength are shown in Fig. 3(c). The maximal modulation depths are 7% at 940 nm, 13% at 660 nm, 8% at 530 nm, and 2% at 455 nm. The measured optical properties of the static top-layer silicone phantom were found to be $\mu_{a}=0.12\;{\mathrm{cm}}^{-1}$, $\mu_{s}^{\prime }=10.2\;{\mathrm{cm}}^{-1}$ at 660 nm, and $\mu_{a}=0.1\;{\mathrm{cm}}^{-1}$, $\mu_{s}^{\prime }=6.5\;{\mathrm{cm}}^{-1}$ at 940 nm, close to the targeted values.

### 3.2 Photoplethysmography Signals Synthesis

**Fig. 4:**
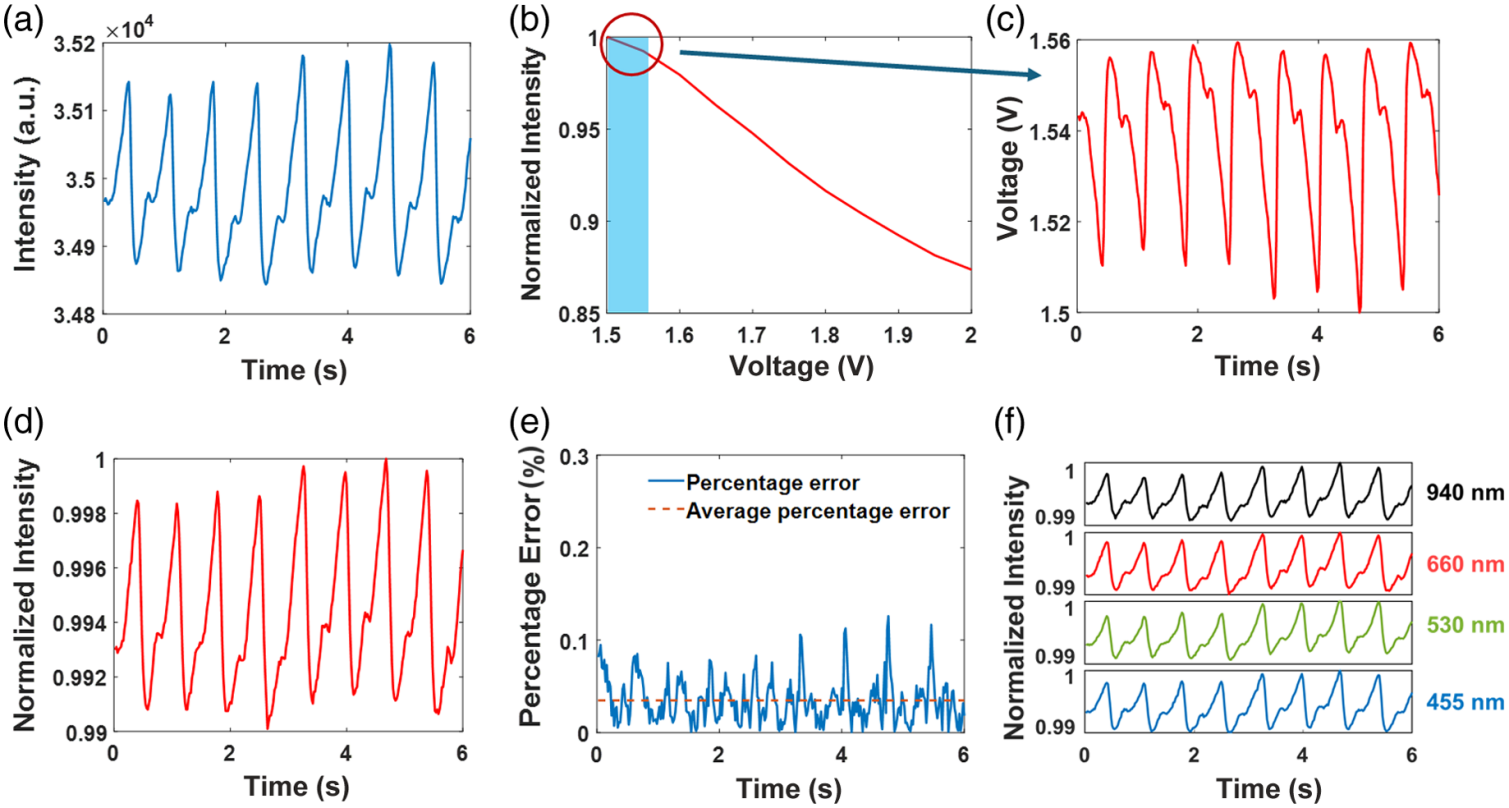
(a) Target PPG signal from the literature. (b) Normalized diffuse reflectance response at 660 nm. (c) Modulation signals for LCD at 660 nm to generate PPG signals. (d) PPG signals generated by the dynamic phantom and measured at 660 nm. (e) Percentage error between target and measured signal after max normalization of the target signal and the average percentage error. (f) PPG signals generated by the dynamic phantom and measured at 940, 660, 530, and 455 nm.

Next, we demonstrate the capability of the dynamic phantom to replicate a targeted PPG trace using 660 nm as an example. Figure 4 shows the steps taken to generate a PPG trace at 660 nm, using the above-mentioned experimentally measured trace from Ref. 31 as a target [Fig. 4(a)]. The normalized diffuse reflectance response within the functional range of the dynamic phantom at 660 nm is shown in Fig. 4(b). The resulting modulation signal required for the LCD to generate the targeted diffuse reflectance from the dynamic phantom is presented in Fig. 4(c). This trace is generated by mapping the normalized original signal to the generated lookup table. Figure 4(d) shows the reproduced PPG signals measured by the photodiode at 660 nm, whereas the percentage error and average percentage error between the target signal [Fig. 4(a)] and the measured signal after max normalization of the target signal are shown in Fig. 4(e). The average percentage errors are 0.03% at 940 nm, 0.03% at 660 nm, 0.05% at 530 nm, and 0.3% at 455 nm. Measured diffuse reflectance traces at four wavelengths are given in Fig. 4(f). The results demonstrate that the dynamic phantom can successfully simulate PPG signals at multiple wavelengths.

To demonstrate the phantom’s ability to generate dynamic biological signals, two case studies were conducted to generate PPG signals of different heart rates and oxygen saturation levels at multiple wavelengths.

### 3.3 Case Study 1: PPG Signals of Different Heart Rates

**Fig. 5:**
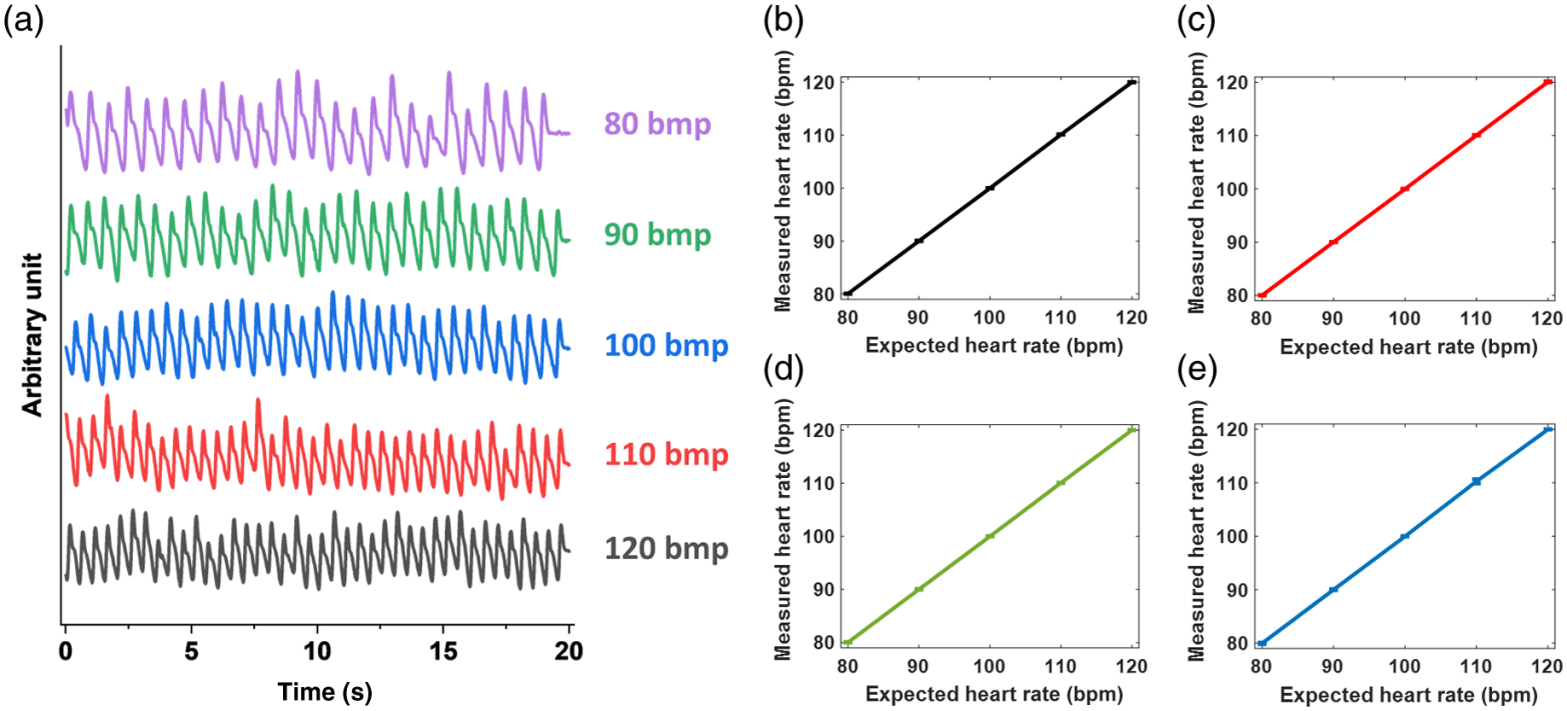
(a) Offset plot of measured signals at 940 nm. Expected versus measured heart rate at (b) 940 nm, (c) 660 nm, (d) 530 nm, and (e) 455 nm.

Figure 5(a) shows 20-s PPG-like signal traces measured from the dynamic phantom for heart rates of 80, 90, 100, 110, and 120 bpm at 940 nm. Each trace shows clear periodicity corresponding to the designated heart rates, indicating the ability of the dynamic phantom to realistic cardiac rhythms. Figures 5(b)–5(e) compare the expected heart rates to the measured heart rates at 940, 660, 530, and 455 nm. The $R^{2}$ values of the linear regression at these four wavelengths are 0.999. The good agreement between the expected and measured values validates the accuracy of the dynamic phantom in replicating various heart rates, demonstrating its potential for calibrating and validating heart rate monitors.

### 3.4 Case Study 2: PPG Signal of Different Oxygen Saturation Levels

**Fig. 6:**
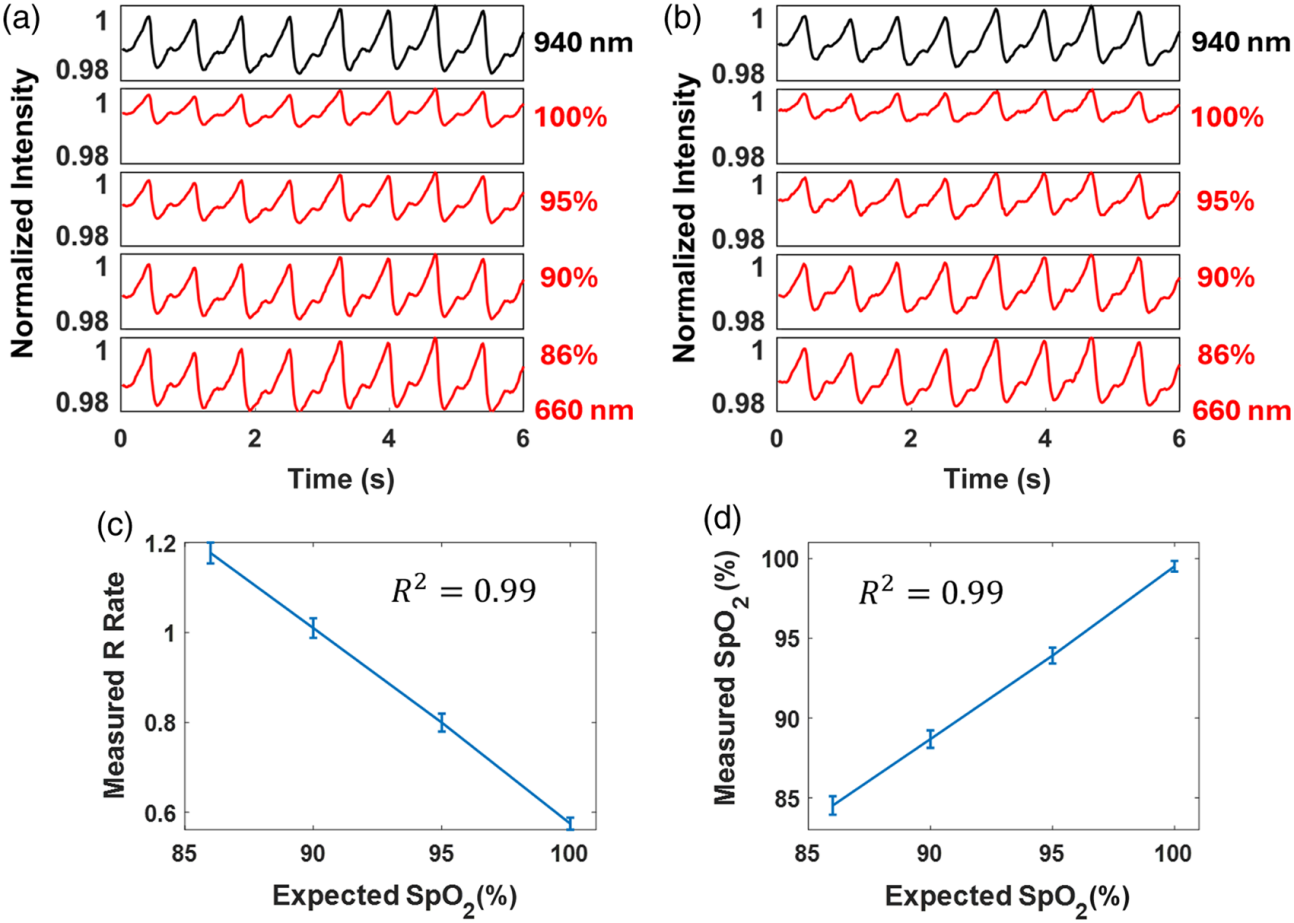
(a) Target signals at different oxygen saturation levels. (b) Measurement results at different oxygen saturation levels. (c) Expected ${\mathrm{SpO}}_{2}$ versus measured R value. (d) Expected ${\mathrm{SpO}}_{2}$ versus measured ${\mathrm{SpO}}_{2}$.

Figure 6(a) shows the target signals at different oxygen saturation levels. Figure 6(b) illustrates the measurement results at different oxygen saturation levels. The measured signals demonstrate clear amplitude variations that correlate with the specified oxygen saturation levels. Figure 6(c) presents the measured R-value as a function of the expected ${\mathrm{SpO}}_{2}$ levels, indicating a linear decrease in the R-value with increasing SpO2. This result aligns with the conclusion of Ref. 35, which states that the R-value should be 1.12, 0.96, 0.76, and 0.55 at 86%, 90%, 95%, and 100% oxygen saturation levels, respectively. Figure 6(d) shows the measured ${\mathrm{SpO}}_{2}$ against the expected ${\mathrm{SpO}}_{2}$, confirming the accuracy of the dynamic phantom in simulating realistic ${\mathrm{SpO}}_{2}$ levels. These results validate the capability of the dynamic phantom to accurately mimic hemodynamics signals and its potential application in calibrating and validating pulse oximeters.

## 4 Discussion

Two case studies mentioned above demonstrate the ability of the dynamic phantom to generate PPG signals at different wavelengths and different oxygen saturation levels. For the characterization of the phantom, the functional range and contrast vary for different wavelengths due to the wavelength-dependent performance of the LCD. The operational wavelength range of the dynamic phantom is thereby determined by the specifications of the LCD. In this setup, the LCD provides broadband visual to near-infrared (400 to 2000 nm) operation with high open-state transmittance optimized for wavelengths around 1100 nm. Consequently, the LCD response shows a larger contrast at 940, 660, and 530 nm at 455 nm. It is also worth noting that only a small fraction of the dynamic range of the functional voltage range has been used to generate the modulation depth in the targeted PPG trace. The maximal modulation depth at 455 nm is 2%, whereas the perfusion index of the PPG signal from a health volunteer is around 1%.^31^ The noise levels are similar at all wavelengths when simulating a 1% perfusion index. The modulation frequency of the phantom is limited by the operation rates of the LCD (1000 Hz) and NI DAQ (400 kHz). In this study, a 50-Hz modulation frequency was used to match the sampling rate in Ref. 31. However, with an upper modulation frequency limit of 1000 Hz, the device can simulate any feasible human heart rate. This allows accurate reproduction of PPG pulse shape features, including the systolic peak, diastolic peak, and dicrotic notch. These capabilities highlight the phantom’s potential to simulate diverse PPG signal shapes for pulse shape analysis in cuffless blood pressure monitoring applications.^36^ In addition to the PPG signal, other dynamic variations in biological tissue, such as hemodynamics, can be simulated by applying the corresponding modulation signals. Compared with the phantom described in Ref. 24, which only characterizes spatial differentiation in attenuation for optical topography, our phantom demonstrates the ability to gradually modulate attenuation with a 153  μV step size voltage modulation and simulate realistic biological signals, making it a more flexible tool for various biomedical applications.

When simulating the PPG signals, various noise signals can be added to simulate motion artifacts introduced by physical movement during the measurement or low-intensity signals due to low blood perfusion. If the phantom is tested using a commercial pulse oximeter, the transmission configuration should be used instead of the reflection configuration. Also, the illumination and phantom modulation must be synchronized as the typical pulse oximeter quickly switches between two wavelengths.^32^ This can be achieved by having a sync input from the pulse oximeter to the phantom, making it possible to dynamically change the phantom modulation to the corresponding wavelength of the pulse oximeter. The other approach is to have two individual light guides and LCDs, one for 660-nm modulation and the other for 940-nm modulation, separately.

In addition to generating dynamic signals, this setup could be used to characterize the spatial resolution of imaging or spectroscopy systems by varying the diameter of the light guide. Multiple light guides can be integrated to create specific patterns to mimic complex biological structures, such as vascular systems. There is no minimum space requirement between neighboring light guides. Compared with the thermochromic dynamic phantom described in Ref. 26, which has a target size of 4.5 mm and requires 11.2-mm spacing between targets, and the phantom presented in Ref. 25, which only provides macroscopic absorption changes, our phantom shows better potential for simulating small and structured biological features with various target sizes. In future research, we intend to incorporate an additional thin-layer phantom with varying melanin concentrations to create a multilayer phantom. This multilayer phantom would mimic different skin colors, facilitating the characterization of skin color bias in pulse oximeters.^37^ The phantom’s low cost makes it widely applicable for both industrial and academic research. The estimated bill of material cost of our demonstration setup is ∼5000 euros, although this may vary depending on the specifications of the LCD and NI DAQ. Currently, the dynamic phantom cannot simulate vascular compliance during the cardiac cycle due to its fixed geometric structure. To address this limitation, future enhancements could incorporate fiber bundles to mimic the vascular structure. The geometry of the dynamic area of the phantom can be controlled by activating or deactivating specific fibers inside the fiber bundle.

## 5 Conclusion

In this study, we have reported the design, characterization, and potential applications of a dynamic phantom capable of generating dynamic biological signals. The absorption properties of the phantom can be controlled in real time by modulating the LCD. The phantom was characterized at four wavelengths: 940, 660, 530, and 455 nm. This dynamic phantom can simulate various biological signals by applying corresponding modulation signals. Clinically relevant case studies were conducted to prove the ability to generate PPG signals of different heart rates and oxygen saturation levels. This dynamic phantom shows the potential of calibrating and validating pulse oximeter, imaging, and spectroscopy systems.

## Data Availability

The code used in this study is available on GitHub: https://github.com/Hui0930/Dynamic-phantom.git.
